# Thinking about translational medicine and traditional Chinese medicine

**DOI:** 10.1186/1479-5876-10-S2-A33

**Published:** 2012-10-17

**Authors:** Boli Zhang, Junhua Zhang, Jingqing Hu

**Affiliations:** 1 China academy of Chinese medical science, Beijing, China; 2 Tianjin University of Traditional Chinese Medicine, Tianjin, China

## 

Translational medicine is a new concept developed in the past two decades. At present, translational medicine has become popular in China and has been introduced into Traditional Chinese Medicine (TCM). In my opinion, putting translational medicine into TCM should consider the characteristics, problems and needs of TCM.

## 1. Translational medicine in TCM is different from western medicine

TCM has a unique theoretical system and mainly reflects the characteristics of the experience-based medicine. The development model of TCM is “from clinical practice to theory and then clinical practice". Experience accumulated from thousands of years’ practice is a great treasure house. A lot of effective herbal medicines and therapeutic methods have been widely used in clinical practice. Because of these, the orientation, contents and mission of translational medical research in TCM and western medicine are different. The strategy of translational medical research in TCM should be “standing on the earth and then achieving the sky”: use modern technologies and methods to solve practical problems in clinical diagnosis, treatment and drug development of TCM; and then establish related standards to leading the development of TCM; finally TCM can be used at a higher level, a wider range in health services.

## 2. Enhance clinical translational research, standardize the treatment protocol and then improve the clinical services level of TCM

TCM clinical practice is based on the theory of overall concept and ‘*Bianzheng lunzhi’.* TCM is a typical individualized medicine which is an advanced developing direction of modern medical science. However, more flexibility than standardization, poor repeatability and lack of high quality evidence are the key issues hinder the clinical service level of TCM. Therefore, the critical task of translational medicine in TCM is to translate the achievements of medical research into clinical practice, to establish a series of clinical diagnosis and treatment technical standards, guidelines and/or pathway which are scientific, generalizable and acceptable by both TCM and western medicine practitioners. The evaluation and validation of the safety and efficacy of Chinese herbal medicine, prescriptions and treatment techniques are key points. Translating the individual experience to the group law can promote the clinical service capability and level of TCM. In addition, the clinical evidence from translational research provides a robust basis and direction for basic research.

## 3. Do translational medical research around key nodes of Chinese medicine R&D, and promote the modernization development of TCM

From active ingredient screening to structural modification, from cell evaluation to clinical trial, target-oriented drug R&D has faced great challenges: low success rate, high cost and high risk. Chinese medicine R&D is not necessary to and should not copy the model of western medicine. Creating new drugs from classic and commonly used prescription, especially the secondary development of marketed Chinese Patent Medicine, is an important content of translational medical research of TCM. Unclear clinical indication, obsolete manufacturing process, unclear material basis and action mechanism, low-level quality control and poor intellectual property protection are the problems more or less existed in the Chinese medical preparations. Using modern technology to solve these problems can achieve the seamless transformation of research results. There are several typical successful examples of translational medical research in TCM. For example, the successful development of artemisinin, the mechanism discovery of arsenic for treating leukemia and Compound Danshen Dripping Pills completed phase II clinical trials in the USA.

## 4. Strengthen the multi-disciplinary cooperation; cultivate translational medicine research team of TCM in practice

Translational medicine is a new medical concept, but no proprietary research methods. Translational medical research of TCM not only needs the knowledge of TCM but also multi-disciplinary technologies and methods, such as biology, chemistry, information science, pharmacology, statistics, epidemiology, drug economics, etc. Paying more attention to the exchange and integration of multi-disciplinary will improve the efficiency of translational research and generate more achievements. To promote the collaboration of universities, institutes and pharmaceutical companies and establish postdoctoral research sites or postgraduate courses for company workers which not only can transform the research results to solve the practical problems but also can cultivate a number of outstanding researchers who are familiar with both company requirement and research methods.

Introducing the concept of translational medicine into TCM, it will take some time from understanding to practice. Translational medical researches should focus on the “need” in practice.

